# Towards evidence-based practice in the social services and older people care: from the line managers’ perspective

**DOI:** 10.1186/1472-6963-14-S2-P82

**Published:** 2014-07-07

**Authors:** Rebecca Mosson, Henna Hasson, Lars Wallin, Ulrica von Thiele Schwarz

**Affiliations:** 1 Medical Management Centre (MMC), Department of Learning, Informatics, Management and Ethics, Karolinska Institutet, Stockholm, Sweden; 2 Centre for Epidemiology and Community Medicine (CES), Stockholm County Council, Sweden; 3 School of Education, Health and Social Studies, Dalarna University, Falun, Sweden; 4 Department of Neurobiology, Care Sciences and Society, Karolinska Institutet, Stockholm, Sweden

## Background

Leadership is essential for successful implementation of evidence-based practice (EBP) [1]. Line managers, i.e. the managerial level directly above employees, are crucial in this process. However, little empirical research has been carried out on their role in the implementation of evidence within social care. The aim of this study was to explore the role of line managers in the implementation of EBP in the social services and older people care.

## Materials and methods

Interviews were carried out with a total of 28 line managers within social and older people care services in seven Swedish municipalities (the local authorities responsible for the provision of these services). A purposeful sampling was performed to ensure diversity among the municipalities in terms of size, geographical location and previous experience with EBP. The interviews were analysed with thematic analysis by two of the authors independently.

## Results

Line managers perceive their role as key when implementing EBP. The extent to which they felt responsible for the implementation process did, however, differ between the social services and older people care. Line managers working within social services portrayed a more positive attitude towards and a more active role in implementing EBP compared to line managers in older people care. Overall, managers working within the social services were generally given more authority from senior management to implement changes in practice. Line managers within older people care were seldom involved in any decision-making concerning implementation of EBP and described their role as solely communicating decisions to their staff. The line managers in both care settings felt alone in the implementation of EBP, and received limited support from the other key actors in their organisation. The implementation process was usually performed ad hoc rather than systematically. Analysis of needs and goals according to the local context were rarely explored.

## Conclusions

Line managers consider themselves as central in the implementation of EBP. Variations exist concerning how line managers in the social services and older people care view and implement EBP, and thus different types of support in improving working evidence-based is required. This research contributes to understanding the perspective of line managers, who are often responsible for the translation of evidence into practice.
